# Interprofessional Education: Medical Students Create a Cadaver Lab Workshop for Nursing Students at a Neutral Cost

**DOI:** 10.7759/cureus.16830

**Published:** 2021-08-02

**Authors:** William Pruitt, Mary Parianos, Nicholas Faraci, David Heaner, Daniel Topping, Joyce Burr

**Affiliations:** 1 Anatomy, University of Central Florida (UCF) College of Medicine, Orlando, USA; 2 Internal Medicine, University of Central Florida (UCF) College of Medicine, Orlando, USA; 3 Nursing Education, University of Central Florida (UCF) College of Nursing, Orlando, USA

**Keywords:** ipe, interprofessional education, nursing, anatomy, cadaver, procedure, student, medical device

## Abstract

Cadaver labs are one of the staples of medical education in the United States, yet it is relatively uncommon for nursing students to have the opportunity to engage in the direct observation, hands-on learning, and the efficiency of the immersive environment in a cadaver-based anatomy lab. The primary aim of this project was to determine if medical students could create and deliver a cadaver lab workshop for nursing students that would provide educational benefits to both groups of students at a neutral cost. The purpose of this activity was to evaluate how a cadaver lab for nursing students could increase understanding of clinically-relevant anatomy, disease, and indwelling medical devices, while enhancing overall clinical problem-solving skills. The participants, nursing and medical students, completed a five-hour workshop followed by completion of a four-question survey of their overall learning experience and the value of the workshop from an interprofessional perspective. The surveys were analyzed individually for qualitative central themes; similar central themes were compiled by question, and overarching themes were identified and reported. Self-reflections completed by the students revealed that this shared encounter between trainees resulted in a better understanding of the visualization of the size, spatial relations, and physical interactions between organ systems; increased confidence in patient care regarding the physical exam and medical device management; and a better-perceived understanding of each profession’s approach in providing patient-centered care. Medical students may also benefit by participation in this interprofessional activity through the development of clinical teaching skills that are needed while working with patients and clinical colleagues.

## Introduction

While many advancements in technology have greatly enhanced a student’s ability to study anatomy, it is the authors' perspective that the direct visualization and hands-on learning that occur in a cadaver lab is difficult to replace. The cadaver-based anatomy lab is a valuable learning experience that is not often available to nursing students in the United States. In a 2014 survey, the National League for Nursing reported 1869 nursing programs in the United States, yet it is relatively uncommon for nursing schools to utilize cadavers for their students’ learning [1].

The aim of this project was to evaluate the University of Central Florida's unique partnership between the College of Medicine and Nursing as one of the relatively few schools in the United States where Bachelor of Science in Nursing (BSN) students could participate in a one-day hands-on session to experience and apply the anatomical reality of what they have learned through models and books. This session was designed so that second- and third-year medical students, who recently completed a cadaver-based gross anatomy course, could teach clinically relevant anatomy to student nurses, while guiding them through clinical scenarios of common pathologies. An additional goal of the workshop was to expose the nursing students to common medical devices in the cadavers from both an external and internal view. 

Potential benefits for nursing students

In a review of anatomy curricula, the best-suggested approach to effective anatomy education is to allow students to use multiple educational modalities, including cadavers [2]. Studying anatomy on cadavers is shown to improve comprehension of spatial orientation of organs and tissues within the body, aid in the development of reasoning skills needed in the clinical setting, and foster humanism and ethics, all of which are all beneficial in the various areas of clinical practice [3,4]. While advanced medical imaging can provide an in-depth internal view of the human body, anatomy dissection allows the student to engage in tactile learning and to appreciate the consistency of the tissue, which cannot be achieved with medical imaging alone [5]. Discussion of pathophysiology and medical devices at the bedside of a body donor would give the nurse the opportunity to have direct visualization of common pathology while working through these scenarios. Additionally, the hands-on aspect of the placement and manipulation of medical devices in the cadaver allows for learning in a low-stress and low-risk environment.

Benefits for medical students

The benefit to medical students in this project is primarily a result of teaching experience and further exposure to anatomy. Currently, at the University of Central Florida, medical students have a 17-week cadaver-based module in their first year and would not have additional exposure to body donor anatomy until an optional elective during their third or fourth year. While third- and fourth-year medical students will have much exposure to assessing and working with living patients in clinical rotations, an anatomy review on cadavers would allow them to apply and visualize new pathology in a hands-on setting.

Medical students would also have their first opportunity to teach peers in a clinical setting. According to Bene and Bergus, there is evidence to show that peer teachers increase their understanding of the content they are teaching by the act of teaching alone [6]. Another study suggests that medical students engaged in teaching activities develop skills to be better prepared for teaching junior physicians and patients during residency training, and medical students who learn teaching skills may become better learners themselves [7,8]. 

Interprofessional education

A shared educational experience between learners of both disciplines would allow nursing and medical students the opportunity to benefit from interprofessional education (IPE). Physicians and nurses work together in the daily care of patients. Good interprofessional teamwork stems from an individual’s understanding and acceptance of the patient care approach of other healthcare specialties [9]. Interprofessional workshops between medical students and nurses help to improve relationships and reduce negative stereotypes between professions, even when the workshop was not designed for this purpose [10]. A subsequent study revealed multiple positive arguments for having nursing and medical student IPE events, including the positive effects of students sharing knowledge and reaching mutual understandings within the context of clinical scenarios [11]. Bi-directional teaching between nursing and medical students demonstrated that shared education in the anatomy laboratory may help positively restructure preconceived attitudes between healthcare trainees of both fields, allowing for effective and collaborative care to predominate in the current and future clinical settings [12].

Resources and potential cost

Some limiting factors that prevent nursing students from being able to participate in cadaver labs are limited access, cadaver preparation, cost of resources, and availability of cadaver-based anatomical educators. This experience was created without any grant requests or financial support from the university by utilizing resources readily available at a typical medical school.

This article was previously presented at the 10th Annual Medical Student Research Conference at the UCF College of Medicine on February 21, 2019.

## Materials and methods

Concepts of curriculum 

Curriculum design was composed of four fundamental concepts: anatomy, pathology, medical devices and clinical scenarios, to help deliver the material in an organized and approachable fashion. Special attention was paid to create a curriculum that would provide nursing students information that would enhance their knowledge in a way that might improve patient care within the scope of nursing practice. The areas of focus were determined by the previous nursing experience of two of the participating medical students. 

Anatomy 

Design of the anatomical curriculum was focused on structures most relevant to the scope of nursing practice (see Table 1). All major structures and organs in the chest and abdomen and their corresponding vasculature were reviewed on cadavers during the lab. Anatomical diagrams and radiographic images were accessed via computers at the bedside of the body donor, so that students could relate diagrams and medical images to what was visualized directly in the body. During the exploration of the chest and abdomen, nursing students were invited to consider whether their physical assessment would change after seeing the spatial orientation of organs as they lay within the body. 

**Table 1:** Anatomy reviewed during the workshop.

Body system	Cadaver anatomy
Musculoskeletal	Outer chest wall, Pectoralis major and pectoralis minor, Psoas major, Psoas minor, Iliacus, Quadratus lumborum, Ribs (including the neurovascular bundle), Vertebral bodies of the spine
Respiratory	Right and left lung (noting the lobes and fissures), Trachea, Bronchi, Hilum of the lungs (noting the pulmonary arteries and pulmonary veins), Pleura
Cardiovascular	Heart chambers (atria and ventricles), Heart valves (tricuspid, pulmonic, mitral, aortic), Coronary artery vasculature, Papillary muscles and trabeculae, Pericardium, Aortic arch, first three branches of the aorta (brachiocephalic, left common carotid, left subclavian), Superior vena cava
Other thoracic cavity structures	Esophagus, Vagus nerve (including the recurrent laryngeal nerve), Phrenic nerve, Sympathetic chain, Thoracic duct, Diaphragm
Gastrointestinal	Stomach (cardia, fundus, body, antrum, pylorus), Small Intestine (duodenum, jejunum, ileum), Colon (cecum, ascending, transverse, descending, sigmoid, rectum), Appendix
Hepatobiliary	Liver (right lobe, left lobe, caudate lobe, quadrate lobe), Pancreas, Gallbladder, Common bile duct, Portal vein
Genitourinary	Kidneys, Ureters, Bladder, Adrenal glands, Prostate, Uterus (fundus, body), Fallopian tubes, Ovaries
Abdominal/pelvic vasculature	Arterial system: Aorta (descending, thoracic, abdominal), Celiac trunk, Superior and inferior mesenteric, Renal, Iliac (common, internal, external), Common femoral; Venous system: Inferior vena cava, Portal, Hepatic, Renal, Iliac (common, internal, external), Common femoral

Pathology 

The approach to delivering concepts of pathology was to screen all available body donors in the lab for disease visible to the naked eye, and then select those that displayed common diseases or dysfunction relevant to nursing practice at the bedside. Enabling nursing students to have hands-on, tactile learning opportunities may allow them to have an elevated appreciation of diseases such as calcified atherosclerosis, nodular hepatic cirrhosis, lung cancer, or diverticulosis. For pathologies that were not viewable from a macroscopic standpoint, the pathophysiological concepts were discussed during the anatomical review of that organ system. For example, renal stones were not viewable or palpable during the workshop, but while viewing the urological system, renal calculi were discussed with reference to common obstructive sites, and what the corresponding effect may be on the ureter and kidney (see Table 2 for specific pathology covered in the workshop).

**Table 2:** Pathology reviewed on the cadavers and via imaging during the workshop.

Body system	Cadaver pathology	Radiographic pathology
Respiratory	Lung mass (malignancy), Emphysema, Anthracosis	Pneumonia, Pneumothorax, Atelectasis, Pleural effusion
Cardiovascular	Coronary artery bypass graft (CABG), Cardiac pacemakers, Mechanical valves, Atherosclerosis of aorta, Cardiomegaly, Left ventricular hypertrophy	Pericardial effusion/cardiac tamponade, Atherosclerosis of aorta, Aortic aneurysm
Hepatobiliary	Liver cirrhosis, Liver mass (malignancy)	Liver cirrhosis, Liver mass
Genitourinary	Atrophic kidneys, Kidney cysts, Benign prostatic hyperplasia	

Medical devices

The medical devices selected in this project included those commonly used at the bedside by nursing, with high utility in critical care scenarios, or that have high risk for dangerous complications (see Table 3). The devices were placed in the body donors in a way that allowed nursing students to view the devices both externally and internally, providing the ability to see the direction and orientation a device takes after passing through the skin. This is beneficial given that nurses commonly are the dedicated first assistant at the bedside when placing devices such as central lines and chest tubes, among others. Visualization of medical devices in the human body may aid these future nurses to better assist their physician colleagues when placing devices and to have more confidence when performing nursing procedures such as peripheral IV insertion, tracheostomy care, or NG tube placement. Nursing students were permitted to engage in hands-on learning with each of the medical devices in the lab and were prompted with hypothetical scenarios, challenging them to troubleshoot a device that was not working at the bedside.

**Table 3:** Medical devices demonstrated on the cadavers or discussed during the workshop.

Body system	Medical devices
Respiratory	Tracheostomy, Endotracheal tube, Chest tube
Cardiovascular	Cardiac pacemaker, Femoral intravenous line, Jugular intravenous line, Peripheral intravenous lines, Radial arterial line, Chemotherapy port
Gastrointestinal	Percutaneous endoscopic gastrostomy (PEG) tube, Nasogastric (NG) tube
Urinary	Foley catheter

Clinical scenarios

Problem-based learning is a staple in many disciplines within health science education. This approach was applied by providing clinical scenarios at the bedside of a body donor. Nursing students were asked to solve these clinical scenario challenges, while having the unique advantage and perspective of looking directly at the circulatory system, pulmonary anatomy, and abdominal viscera. Clinical scenarios presented included those where early recognition is paramount for patient outcome (see Table 4). All major forms of shock were reviewed, as well as multiple hypothetical airway challenges, such as troubleshooting a clogged inner cannula of a tracheostomy or a dislodged outer cannula. 

**Table 4:** Clinical scenarios discussed with students during the workshop.

Body system	Clinical scenarios
Respiratory	Accessing an airway through endotracheal intubation, Proper usage of a bag valve mask, Tracheostomy and cricothyrotomy placement, Proper tracheostomy care, Pulmonary embolism, Chest tube placement
Cardiovascular	Gaining vascular access through a peripheral and central intravenous line, Gaining intraosseous access, Proper chest compression placement and technique, Needle placement for cardiac tamponade decompression, Myocardial infarction/cath lab procedure, Types of shock
Gastrointestinal	Providing enteral nutrition, Ruptured esophageal varices, Esophageal rupture, Ruptured diverticulum, Ischemic bowel, Peptic ulcer disease, Intestinal perforation, Appendicitis
Hepatobiliary	Cholecystitis, Pancreatitis, Pancreatic cancer, Stigmata of liver cirrhosis
Genitourinary	Foley catheter placement, Urinary tract infections in the elderly, Pyelonephritis, Hydroureter/hydronephrosis, Bladder scanning, Tubal ligation, Hysterectomy

A study by Mc Garvey et al. revealed that nursing students experience increased levels of stress when working with body donor tissue for the first time when compared to plastic anatomical models [13]. To reduce stress and anxiety, the nursing students were provided with College of Medicine approved cadaver lab video documentaries that discussed the history of cadaver labs and the emotional impacts that it may have on the student. Pre-lab and post-lab briefing sessions were conducted to discuss emotional impacts that students commonly experience in anatomy labs; this allowed time for students to discuss any thoughts or concerns.

Day-of-workshop instruction

On the day of the workshop, nursing students were divided into two groups with a ratio of five to six nursing students to two medical students. Two cadaver stations were organized: one demonstrating the thoracic cavity and the other the abdominal cavity. Nursing students received one hour of interactive anatomy, pathology, and clinical scenario instruction at each station. After completing their first station, the groups alternated. If the discussed abnormal pathology was available to view on the cadavers presented or any additional cadavers in the lab, this was demonstrated to the students; otherwise, the remaining pathology was discussed via imaging as a hypothetical with the cadaver for reference. After all nursing students had completed both the thoracic and abdominal stations, they then participated in two thirty-minute medical device stations, where students received instruction on the medical devices and hands-on practice with select devices. Two additional body donors were used for the medical device demonstration. The devices were inserted by medical students prior to the day of the lab. In the final hour of the lab, students were given time for self-directed hands-on learning on all of the body donors, and medical students were available to answer questions. The students were provided with self-reflection questions one week after completion of the lab. This workshop was performed twice with a total of 22 nursing students (10 students and 12 students, respectively).

## Results

Open-ended qualitative surveys were provided to the nursing students to evaluate the success of the cadaver lab. Informed consent was obtained from the students, and all participation in this study was voluntary. This study was reviewed by the local University of Central Florida (UCF) IRB and was exempt from IRB approval as it was determined to be nonhuman subject research (IRB #STUDY00001729).

The survey was provided the nursing students across both workshop experiences. It contained the following four questions: 

1. How has your understanding of humans/humanity changed? 

2. What are your impressions of the value of interprofessional learning? 

3. Would you recommend this experience to others -- why? 

4. What would you like to share that you have not been asked? 

The surveys were individually assessed for central themes; these themes were compared to one another to identify the most common themes across the surveys. The common themes appreciated from this analysis include an overall positive experience for the students, expression of the importance of a cadaver lab in healthcare education, better appreciation and understanding of human anatomy, clearer understanding of disease processes on the body, and the importance of interprofessional learning as a means to increased respect and understanding of other disciplines. 

As for the importance of an anatomy lab in education, the students specifically noted that the ‘hands-on’ aspect of the lab allowed for increased understanding of spatial organization within the human body as well as appreciation for organ size. It was noted that this led to increased self-confidence for the students with physical exam skills, handling medical devices, and patient education. Additionally, several students felt that their experience in a cadaver lab led to better retention of information in comparison to other methods of anatomy education. 

Many students remarked on the importance of interprofessional education. The type of learning environment fostered by allowing medical students to host the anatomy lab for nursing students, with both groups sharing their knowledge and clinical experience, was found to be well-received and appreciated. In the words of one of the nursing students, this created a “professional low-stress environment.” It was observed by the students that interprofessional learning leads to a better perspective of other health disciplines, as well as increased respect for other disciplines. It was also noted by the students that interprofessional learning is an excellent foundation for improved future interprofessional partnership. 

In review of the surveys given to the medical students, it appears that very similar themes were present. The medical students remarked that this workshop not only allowed them to review relevant anatomy and physiology prior to clinical rotations, but also further developed their clinical foundation through the teaching and knowledge sharing experiences that occurred between the medical and nursing students. The medical students also noted the lack of medical device experience in medical education and appreciated the opportunity to learn about them and their placement alongside the nursing students. Several positive themes were identified regarding interprofessional education, with a better understanding of nursing practice and the opportunity to develop professional friendships and relationships noted most. 

A compilation of the common themes with examples of direct student quotes can be seen in Table 5 and Table 6. 

**Table 5:** Themes and quotes from nursing student survey one week after the workshop.

Main themes	Survey quotes
Positive experience	“This should be a mandatory part of the nursing curriculum.” “[This] will be carried with me throughout the rest of my nursing program and into my clinical experience.” “I think this is something all nurses should experience.”
Importance of cadaver lab in education	“Being able to directly interact with the anatomy, along with the background knowledge I have, has given me the ability to better visualize and understand what is going on inside the body.” “[My] didactic education paled in the knowledge I [gathered] from just one experience at the cadaver lab.” “I feel like I retained more in 5 hours of the cadaver lab than I did for an entire semester of [anatomy and physiology] with plastic models [and] pictures.” “The hands-on anatomy isn’t anything a book [could] ever compare to.”
More comfortable providing patient care	“I believe that it will help me visualize what I am palpating… when I am doing an assessment.” “Seeing the invasive procedures performed in a safe, pain free environment provides students the [ability] to further develop skills and increase confidence before real world application.”
Importance of interprofessional education (IPE)	“As nursing professionals, … the ability to develop these teamwork skills early gives us a chance to see healthcare from many different perspectives.” “[IPE] supports the teamwork and collaboration of an interdisciplinary team.”
Better appreciation of the human body	“The ability to see the human body… truly [increased] the value of each human life [for me] and [gave] me an appreciation for each patient that I will care for in the future.” “...newfound appreciation for how delicate and harmonious the body is.”
Better understanding of anatomy	“I have a clearer understanding of how nerves, veins, and arteries actually look and interconnect throughout the human body.”
Clearer understanding of disease processes on the body	“It’s easy to read about how cancer and tumors are abnormal accumulations of cells etc… but being able to touch the organs and feel the tactile differences gave a different appreciation for how devastating these diseases can be.”
Preferred medical students hosting the cadaver lab	“I was ecstatic that the medical students were the ones who taught and guided the lab… it made me less nervous.” “Low stress environment.”
Appreciation for cadaver donors	“This experience has made me deeply consider donating my own body to science.” “[Seeing] just how essential these bodies are for educational purposes was extremely profound. I feel truly grateful for those who have chosen to donate their bodies.”

**Table 6:** Themes and quotes from medical student surveys after the workshop.

Main themes	Survey quotes
Excellent learning experience	“Good review of anatomy in preparation of the lab.” “Involving radiology imaging, medical devices, and clinical scenarios helped to… further my understanding of human anatomy.” “Not only was I an instructor for this activity, but... a learner as well.”
Development of teaching skills	“This activity challenged me to become a better educator as the nursing students often asked tough questions.” “[You] have to think about how you want to communicate information for best effect.”
Opportunity to work with medical devices	“I feel more comfortable with putting devices in at the bedside after some cadaver experience.”
Learning from nursing students	“The nursing students benefited me by teaching me more about the medical devices.”
Importance of interprofessional education	“[Allowed us to] connect with nursing students [and] demonstrate that we want to work together for the good for the patient.” “Building a foundation of interprofessional collaboration.”
Appreciation for cadaver donors	“ I was able to see how one cadaver could affect students from multiple professions and give these students an experience they will never forget.”

## Discussion

Reported benefits for nursing students

The response from nursing students in their open-ended surveys was overwhelmingly positive. While many themes were reported repeatedly, a few themes stood out as ones that furthered the students’ education with relevance to patient care. 

One theme that emerged from the nursing student data was the opportunity to take theoretical and abstract concepts from nursing textbooks and lectures and convert these ideas to more concrete knowledge as they viewed and handled organs and vasculature within the human body. Nursing students reported that this practice helped them to “connect the dots” between textbooks and the human body. The nursing students stated that this new tangible perspective of the human body would positively affect the way they performed physical examinations and also noted that this experience would help with long-term retention of both anatomical and pathophysiological knowledge.

A second theme from the nursing student surveys that have high utility in clinical practice was the frequent mention of a greater understanding of the correct placement and function of medical devices. Nursing students reported increased confidence in performing nursing procedures, such as IV insertion, JP drain removal, and tracheostomy care. Additionally, increased confidence in assisting physicians with bedside procedures, such as central line or chest tube placement, was noted. The participants commented that they feel more prepared to properly use and monitor these devices at the bedside. Multiple students noted that the increased understanding of the internal placement of medical devices will make them feel more prepared to discuss them when providing patient education in the future.

Reported benefits for medical students

The most impactful experience reported by the medical students was the challenge of changing roles from student to educator by designing and delivering curriculum for another discipline in healthcare. One student reported that this process required them “to prepare and think ahead of time how... to communicate information for best effect.” The anatomy lab workshop gave the medical students an early opportunity to develop and practice clinical teaching skills, which will be essential as they progress into their future careers. After a medical student graduates and transitions to residency, one of their main responsibilities is to teach medical students and junior residents. Throughout their careers, physicians educate patients on the many aspects of health, disease, and treatment options. Well-developed teaching skills will have a direct benefit in providing quality patient care. All medical students who participated in the project reported increased confidence in teaching skills and specifically reported increased confidence in their ability to teach nurses, and nursing students, in the clinical setting. An unintended outcome of the workshops is that multiple medical students are now considering careers in academic medicine. 

The medical students reported an increase in their medical knowledge base because of their participation in teaching the workshops. By designing the teaching curriculum, the students were challenged to review anatomy, pathology, and medical devices from a clinical educator perspective. In doing so, they created a structured process to deliver material to nursing students. Multiple participants commented that working together in teams to build the curriculum facilitated learning and furthered their own clinical education. Because the participating second-year medical students were still in the pre-clinical phase of their education, the students found that they were able to learn from the clinical experience of the nursing students. The medical students reported that questions asked by the nursing students challenged them to think of medical topics in a way that they had not considered before, helping to broaden their approach to clinical problem-solving. A further advantage was an opportunity for early exposure to medical devices in their pre-clinical education. The medical students believed that learning about and placing these devices would give them more confidence when seeing, monitoring, and placing devices in the future. 

Reported benefits of IPE

Two frequently mentioned IPE themes by the nursing and medical students were (1) a better understanding of the scope of practice of each profession and (2) an opportunity to create a bond with the other profession that will translate to future collaborations when caring for patients.

Regarding the scope of practice, nursing students reported a better understanding of how focused medical students are on making a diagnosis and deciding on the best treatment options available. Medical students reported a better understanding of nursing practice and discovered that, while nursing students have similar goals, they are also tasked with the challenge of providing safe and efficient care at the bedside. From the perspective of the medical student, a new respect was gained for challenges nurses face, such as a patient’s changing condition or a malfunctioning medical device, and how a nurse must respond in a way that delivers the most appropriate care at the bedside in real-time. Both the nursing and medical students reported that after learning more about the scope of practice of the other profession, they, in return, had a better sense of the scope of practice of their own profession. Medicine and nursing share significant overlap in clinical practice and have frequent interactions, so it is important for both professions to fully understand the scope of the other. Doing so will help ensure that the most complete care is provided to each patient encountered in the future.

Both nursing and medical students mentioned that working and learning together in the workshop was an initiation of professional “bonding” and expressed that “interprofessional education is a bridge to future interprofessional collaboration.” Both student groups thought that collaborative learning during their education may lead to more effective communication, better patient care, and may help reduce the risk of medical errors in the future. Nursing students and medical students reported that working in this “low-stress environment” gave them a chance to communicate more openly and allowed them to freely ask and answer questions during the instructional period. 

Resources and cost**

Prior to the creation of this workshop, the authors proposed that a cadaver lab experience could be created at a medical school for nursing students at a neutral cost. The entire laboratory experience was completed without any grant support or request for funding via utilization of resources that would be commonly available at most American medical schools. At many medical schools in the United States, first-year medical students will complete cadaver dissections as part of their routine education. The authors gained permission to utilize these body donors for the workshop, which already had the thoracic and abdominal cavities dissected by first-year medical students. Using these prosected body donors allowed savings on cost and preparation time. To alleviate the potential high cost of medical devices, the project was discussed with a supply manager at a local university-affiliated hospital, and the hospital donated a number of expired medical devices that were otherwise planned to be discarded. By utilizing qualified second-year medical students as teachers, the increased cost of using paid anatomy professors for this workshop was negated. Through these methods, this workshop was successful without the need for additional spending.

The limitations of this project include the relatively small class sizes that participated in the workshops and the limited five-hour duration of each session. The class size was kept small, as this was a pilot program, and the goal was to assess its utility before offering it to a large group. Another limitation is that the pathologies that could be observed were dependent on the cadavers that were present in the anatomy lab. This limited the number and types of pathologies that could be observed by the students.

## Conclusions

The best approach to effective anatomy education includes the use of a cadaver lab, per previous studies, to which nursing students are infrequently exposed. Through this workshop, nursing students reported a better understanding of human anatomy, a better appreciation for spatial relation and size of organs, and increased confidence with the handling and troubleshooting of medical devices, etc. The medical students noted growth in their teaching skills in addition to reinforcing their knowledge base. Through donations and volunteering by medical students, this workshop was orchestrated at a neutral cost.

Given that the majority of medical schools in the United States are at universities that have nursing schools associated with them, this type of program could potentially be instituted at most medical schools in the country that wish to offer this experience to their students. Future thoughts on this project would be to quantitatively measure improvement via quizzes before and after the workshop or to potentially assess nursing students who have been exposed to this workshop in comparison to nursing students who are trained via textbook, imaging and other means. A potential goal would be to expand this project to a larger group of nursing students within each workshop with a goal of rotating all BSN students through the lab before their graduation. It is recommended to maintain a ratio of two medical students to six nursing students, as this provides the nursing student the ability to consistently ask questions and the medical student access to a co-teacher to aid with the teaching process. Future workshops would also include an expansion of the current curriculum. Based on the responses and recommendations of the participating nursing students, it would be beneficial to add basic intra-cranial anatomy and additional time for the musculoskeletal and vascular anatomy of the upper and lower extremities. Clinical faculty could adopt a more expanded role as formal evaluators for the medical students to help develop their teaching skills for potential future careers in academic medicine.

## References

[1] (2020). National League for Nursing. National League for Nursing.

[2] Estai M, Bunt S (2016). Best teaching practices in anatomy education: a critical review. Ann Anat.

[3] Rizzolo LJ, Stewart WB (2006). Should we continue teaching anatomy by dissection when ...?. Anat Rec B New Anat.

[4] Azer SA, Eizenberg N (2007). Do we need dissection in an integrated problem-based learning medical course? Perceptions of first- and second-year students. Surg Radiol Anat.

[5] Miles KA (2005). Diagnostic imaging in undergraduate medical education: an expanding role. Clin Radiol.

[6] Benè KL, Bergus G (2014). When learners become teachers: a review of peer teaching in medical student education. Fam Med.

[7] Bulte C, Betts A, Garner K, Durning S (2007). Student teaching: views of student near-peer teachers and learners. Med Teach.

[8] Dandavino M, Snell L, Wiseman J (2007). Why medical students should learn how to teach. Med Teach.

[9] Herrmann G, Woermann U, Schlegel C (2015). Interprofessional education in anatomy: learning together in medical and nursing training. Anat Sci Educ.

[10] Lockeman KS, Appelbaum NP, Dow AW, Orr S, Huff TA, Hogan CJ, Queen BA (2017). The effect of an interprofessional simulation-based education program on perceptions and stereotypes of nursing and medical students: a quasi-experimental study. Nurse Educ Today.

[11] Homeyer S, Hoffmann W, Hingst P, Oppermann RF, Dreier-Wolfgramm A (2018). Effects of interprofessional education for medical and nursing students: enablers, barriers and expectations for optimizing future interprofessional collaboration - a qualitative study. BMC Nurs.

[12] Merati N, Murphy-Buske A, Alfaro P, Larouche SS, Noël GP, Ventura NM (2021). Professional attitudes in health professions' education: the effects of an anatomy near-peer learning activity. Anat Sci Educ.

[13] Mc Garvey A, Hickey A, Conroy R (2015). The anatomy room: a positive learning experience for nursing students. Nurse Educ Today.

